# Fragmentation of Contaminant and Endogenous DNA in Ancient Samples Determined by Shotgun Sequencing; Prospects for Human Palaeogenomics

**DOI:** 10.1371/journal.pone.0024161

**Published:** 2011-08-31

**Authors:** Marc García-Garcerà, Elena Gigli, Federico Sanchez-Quinto, Oscar Ramirez, Francesc Calafell, Sergi Civit, Carles Lalueza-Fox

**Affiliations:** 1 Institut de Biologia Evolutiva, CSIC-UPF, Barcelona, Spain; 2 Department de Bioestadística, Universitat de Barcelona, Barcelona, Spain; Natural History Museum of Denmark, Denmark

## Abstract

**Background:**

Despite the successful retrieval of genomes from past remains, the prospects for human palaeogenomics remain unclear because of the difficulty of distinguishing contaminant from endogenous DNA sequences. Previous sequence data generated on high-throughput sequencing platforms indicate that fragmentation of ancient DNA sequences is a characteristic trait primarily arising due to depurination processes that create abasic sites leading to DNA breaks.

**Methodology/Principals Findings:**

To investigate whether this pattern is present in ancient remains from a temperate environment, we have 454-FLX pyrosequenced different samples dated between 5,500 and 49,000 years ago: a bone from an extinct goat (Myotragus balearicus) that was treated with a depurinating agent (bleach), an Iberian lynx bone not subjected to any treatment, a human Neolithic sample from Barcelona (Spain), and a Neandertal sample from the El Sidrón site (Asturias, Spain). The efficiency of retrieval of endogenous sequences is below 1% in all cases. We have used the non-human samples to identify human sequences (0.35 and 1.4%, respectively), that we positively know are contaminants.

**Conclusions:**

We observed that bleach treatment appears to create a depurination-associated fragmentation pattern in resulting contaminant sequences that is indistinguishable from previously described endogenous sequences. Furthermore, the nucleotide composition pattern observed in 5′ and 3′ ends of contaminant sequences is much more complex than the flat pattern previously described in some Neandertal contaminants. Although much research on samples with known contaminant histories is needed, our results suggest that endogenous and contaminant sequences cannot be distinguished by the fragmentation pattern alone.

## Introduction

Ancient DNA (aDNA) analysis on extinct human populations can potentially provide information on past human migrations and evolutionary processes. aDNA extracts are composed, in variable ratios, of endogenous DNA (either damaged or undamaged) and exogenous DNA. The exogenous DNA can include environmental contaminant DNA (mainly DNA from microorganisms such as bacteria) derived from sample exposure in the ground, but also sources that might enter post excavation, e.g. human DNA derived from handling/manipulation of the ancient samples. Several years ago, the adoption of several laboratory practices were advocated as a means to ensure the authenticity of aDNA results [1]. These included, for instance, physical separation of the ancient and modern DNA laboratory, frequent bleaching and UV irradiation on the working surfaces, the use of sterile laboratory wear (including gloves and face masks), cloning of PCR products, and the independent replication of the results in a second laboratory. However, all these authentication criteria have proven to be ineffective while working on ancient human specimens, because modern human contaminant DNA can be mistaken for endogenous DNA [2]. This is due to the near impossibility of discriminating between the two types of sequences, and the fact that not all the contamination can be controlled for within the laboratory, even under the most stringent precautions. Several studies pointed to the existence of pervasive pre-laboratory contamination, as showed by the presence of human DNA in non-human samples, such as pigs, foxes, cave bear, dogs or Neandertals [3], [4]. Therefore, independent replication of the results in another laboratory will not eliminate the problem.

Human handling-derived contamination can to some extent be monitored during excavation, in which the sequences of the people that recently have handled the remains can be genotyped. Studies that have followed this approach have identified significant amounts of these traceable contaminating sequences, mixed with the putative -and usually prevalent- endogenous ones [5]. It has been suggested that ancient human DNA specimens destined for future palaeogenetic studies should be based on material extracted under controlled conditions (e.g., with sterile lab gear and with the immediate freezing of the samples) [5]. Unfortunately however, the majority of human archaeological material available for study today derives from excavations in the past, in which these precautions were not implemented. An additional concern is that, as contaminants age, handling-derived contaminant DNA molecules themselves can be chemically degraded to a level similar to that observed among the endogenous sequences [6], thus rendering it impossible to discriminate between the two types of sequence using characteristics such as postmortem DNA damage alone [7], [8].

A further authentication criterion that has been proposed, that of “appropriate molecular behaviour”, refers to potential differences in length between the endogenous and the contaminant DNA. As time passes following the death of an organism, the surviving DNA is subjected to damage processes that break the double helix and degrade it DNA molecules into progressively smaller fragments [9]. Since contaminant DNA molecules are in general considerably more recent than those endogenous to an ancient sample, it is assumed that the former will often be of larger average size than the original DNA. Mälmstrom et al. [4] analysed dog samples contaminated with human DNA and found more authentic DNA in shorter than in longer fragments retrieved. Thus, ancient samples show an increase in authentic DNA yield with decreased fragment size than the contaminating DNA. This asymmetrical behaviour could be explained if the contaminant sequences are on average longer than the endogenous ones [4].

Recently, high-throughput sequencing techniques, such as 454-FLX pyrosequencing (Life Sciences-Roche) or the Illumina Genome Analyzer platforms have enabled the generation of large numbers of ancient DNA sequences, even resulting in the first complete ancient genome sequences [10]–[13]. The advent of these techniques has necessitated a re-examination of authenticity criteria, in light of the new methods through which data is generated. For instance, it is now possible to directly observe the distribution length of endogenous and contaminant sequences in a shotgun sequenced aDNA extract, for example as has been demonstrated with Neandertal DNA [14]. Under the assumption that short sequences are endogenous, and longer sequences contaminant, this in theory can be used as a means to discriminate contaminant from endogenous sequence. How straightforward it is to do this however is unclear. While endogenous DNA sequences from different DNA extracts have similar distributions (although differing by sample on their modal and average length), the modern human contaminant sequences that have been reported exhibit rather different length distributions among samples. For instance, in one Neandertal specimen (El Sidrón 1253), all modern human contaminants were between 30–60 bp in length, while in others (Feldhofer 1 and Vindija 33.16), they ranged from 30 up to >200 bp [15]. Nevertheless, despite this apparent heterogeneity, the reanalysis of the first published Neandertal genome sequences showed that the longest sequences (accounting for up to 80% of all reads) were likely modern human contaminants [16].

An alternate characteristic of shotgun sequenced DNA that can be analysed, and that can play a role in discriminating between contaminant and endogenous DNA, is the examination of the nucleotide composition patterns near the 5′ and 3′-ends of the 454-FLX generated sequences. For example it was demonstrated that in endogenous Neandertal sequences, guanine and adenine residues are elevated relative to cytosine and thymine residues, immediately before the strand breaks [17]. Additionally, at the 5′-end of sequences, thymines are present at above average frequencies, while at the 3′-end of sequences, adenines are increased, due to a combination of the effects of enhanced rates hydrolytic deamination of cytosine into uracil or its analogues in the 5′ single strand overlaps that are believed to be common in aDNA, combined with the action of T4 DNA polymerase during sequencing library construction [c.f. 17 for full details]. Based upon these observations of DNA damage, it is believed that the primary cause of fragmentation in ancient sequences is DNA depurination [17]. Given the fact that such damage is believed characteristic of aDNA, bioinformatic tools have now been already developed to analyse these characteristic patterns so as to provide a measure of credibility to aDNA results [18]. However in this regard it is worth noting that the observations made so far have been made in samples that share a number of traits, such as being dated to the Pleistocene, having a low or negligible degree of human contamination, and that contain data that allows the phylogenetic discrimination of endogenous and contaminant sequences [13], [15], [19]–[21]. Thus it is important to widen the range of samples that such damage analyses have been undertaken on, the aim of this study.

To further explore whether DNA depurination is the primary mechanism underlying the detected fragmentation pattern, we have analysed modern human contaminant sequences obtained from a 454-FLX sequenced non-human ancient sample that had been treated with bleach prior to DNA extraction, a known depurinating agent. Secondly, we have explored the DNA fragmentation patterns of human contaminant sequences obtained by 454-FLX sequencing a non-bleach treated non-human ancient sample. Thirdly, we have 454-FLX sequenced two ancient human samples from different ages and taphonomic conditions: a recently found Neandertal sample and a Neolithic human sample, to investigate whether the observed sequence fragmentation patterns are consistent with those previously described.

## Materials and Methods

To generate a dataset of known human contaminant sequences, DNA extracts from two different non-human samples were built into MID tagged 454-FLX libraries, then pyrosequenced at the Centre de Regulació Genòmica (CRG) in Barcelona: a *Myotragus* bone and an ancient Iberian lynx mandible. Myotragus balearicus was an extinct endemic caprine from the Balearic Islands [22]. A Myotragus radius bone (IMEDEA 43619) excavated from Cova Estreta (Pollença, Mallorca) in 1996 and radiocarbon-dated to about 6,300-5,700 years ago was analysed [23]. The Iberian lynx (Lynx pardinus) is a critically endangered carnivore currently restricted to two isolated populations in the south of the Iberian Peninsula. However, until historic times its distribution was larger, reaching the North East of the Iberian Peninsula. A lynx mandible from Cova del Toll (Barcelona) dated to about 11,420 years ago, was analysed [24].

To explore the pattern of human sequences found in contaminated ancient (anatomically modern) human specimen, we similarly 454-FLX sequenced DNA extracted from a Neolithic tooth (inventory number CCG94-E33) belonging to the site of “Camí de Can Grau” (Granollers, Barcelona, Spain). This is a necropolis excavated in 1994, that comprised 23 tombs radiocarbon dated to between 5,500-5,000 years ago, and that was previously studied by conventional, PCR-based methods [25]. It is known that these samples have been washed and handled without special precautions by the excavators, and had a contamination content that was quantified using conventional PCR and cloning as about 17.1% of all PCR-produced mtDNA sequences [6]. However, the contaminating sequences in this particular sample (unpublished data) seem to be lower (∼5%) than the average of the other samples from this particular site [6].

In addition we 454-FLX sequenced a Neandertal bone fragment from the El Sidrón site (Asturias, Spain), dated to about 49,000 years ago and labeled SD 1504. Previously, two mitochondrial DNA fragments were determined by PCR from this sample, and no contaminant sequences were found among 115 clones generated (data not shown). Therefore, little or no contamination is suspected to be present within this sample and thus, it we hypothesised that it could be taken as a control from which the ‘true’ endogenous DNA fragmentation pattern could be discerned.

Post sequencing, the following protocol was followed so as to describe the fragmentation patterns among the data. Starting with the raw 454-FLX sequence files, the sequence reads were analysed to check for the presence or absence of both (5′ and 3′) MID adaptor tags. Only the reads containing both adaptor sequences were kept in this analysis, to avoid possible mis-assignment of the sequence ends. The remaining sequence reads were aligned using NCBI Blast with the megablast algorithm, against the non-redundant nucleotide database (nt). Only blast hits with a coverage over 98% were retained, and, in case of uncertainty in the blast assignment, the higher bit score hit was selected. The blast output was assigned to a specific taxonomic level, using the NCBI taxonomy database. TaxID assigned blast outputs were backtracked to the order level, and filtered keeping only the sequences that matched to primate taxa using a customised perl script that performs the Lowest Common Ancestor algorithm (M.G.G, unpublished). To avoid uncertainties in finding the fragmentation point in the sample sequence, the remaining 454 reads were extended 10 nt up- and downstream, using the GI reference sequence and the extreme up-/downstream 21 nucleotides to analyse the fragmentation pattern. Nucleotide frequencies for the resulting 21 positions were calculated.

To sum up the frequency patterns on the fragmentation point of the ancient DNA sequences we used entropy variation as statistical measure. Given the definition of entropy as a measure of the uncertainty associated to a random variable, we can calculate the entropy per position using the Shannon diversity as:
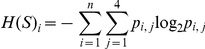
where H(S)_i_ is the entropy of the i-th position of a given dataset of n sequences, and p_i,j_ is the frequency of the j-th nucleotide for that given position, resulting in a single value associated with each position that sums up all diversity information.

Given the low numbers of primate sequences in all datasets, we used a bootstrap method to assess the variation in entropy along the breakpoint. As variability in the fragmentation point tends to decrease in the ancient endogenous sequences, the uncertainty level decreases, and the bootstrap resampling would give us the statistical power to compare the different samples and to check for the level of contamination.

## Results

### Human contaminant sequences in animal bones

**Figure 1:**
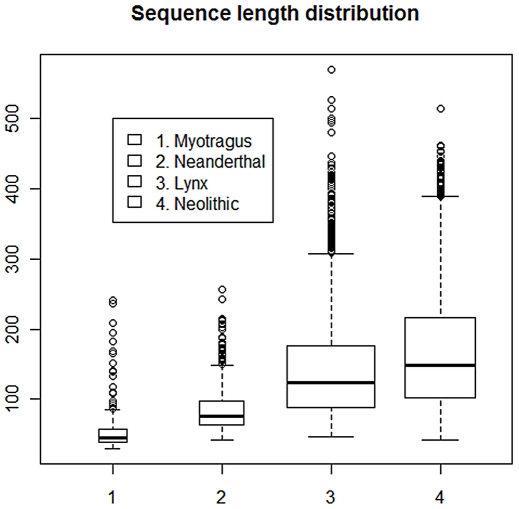
Sequence length distribution of the human contaminant sequences obtained from DNA extracted from bone samples of a *Myotragus balearicus* and an ancient lynx, and the putatively endogenous sequences from a Neolithic human and a Neandertal specimen obtained by 454-FLX pyrosequencing. The contaminant reads in *Myotragus* are possibly fragmented by the bleach treatment; nevertheless, a large overlapping in the distribution length can be observed among samples.

**Figure 2:**
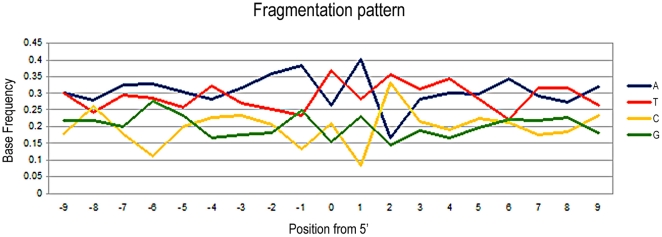
Nucleotide base frequencies at the 5′ end of the *Myotragus* human contaminants, treated with a depurinating agent, bleach. The base composition is plotted as a function of distance from the 5′-end. Despite the small sample size (N = 337 reads), the pattern matches that previously described in ancient endogenous sequences, including Neandertals.

**Figure 3:**
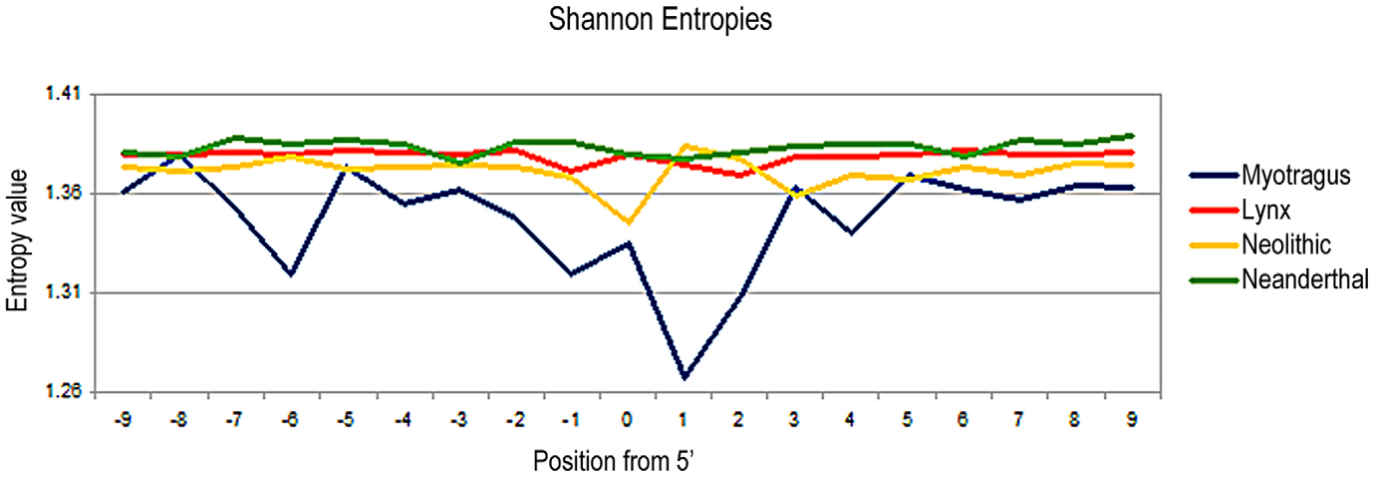
Entropy at the 5′ end of the *Myotragus*, the human contaminants in the lynx, the Neolithic and the Neandertal reads, estimated using Shannon equation and 100 bootstraps. It can be seen that in *Myotragus* and Neolithic the entropy drops at the breaking point, indicating that sequences are not randomly fragmented, while in lynx and Neandertal, the entropy is stable (in the latter this is due to the small sample size of the Neandertal reads available).

Among the 96,357 sequences obtained from *Myotragus*, 337 (corresponding to 0.35% of the total) could be identified as contaminant human sequences (a greater number than the endogenous sequences, that accounted for only 260 reads [0.27%]) (Table 1). These human sequences probably originated from palaeontologists who washed and cleaned the remains after their excavation in 1996, inadvertently contaminating the specimen. The average length of these sequences is 85 nucleotides, and they range from 30 bp (determined by the length cut-off in the analysis) to 300 bp (limited by the 454-FLX technology used) (Figure 1). Filtering to remove reads that did not contain the adaptor at the 3′ end (thus reads derived from a library insert longer than the maximum sequence read length capability) lead to removal of only a small fraction of reads (<1%). This low level renders it unlikely that the subsequent analyses will be biased due to the size distribution of the endogenous versus contaminant reads. Moreover, frequency and entropy calculations at the 5′ end, including the sequences without 3′, did not result in any difference on the results. From the data we note that the length distribution of the human contaminant sequences is similar in shape to previously reported endogenous aDNA distributions that have also been generated by shotgun sequencing methods [14], [19], [20]. Furthermore, the fragmentation pattern in both the endogenous and the human contaminant sequences is concordant with the suggested depurination pattern characteristic of endogenous aDNA [17] (Figure 2), with the entropy clearly decreasing immediately prior to the breakpoint, indicating that fragmentation is not produced at random (Figure 3). Given that the bone powder was treated with bleach prior to DNA extraction as a decontaminating procedure [23], the data suggests that bleach treatment may convey ‘ancient’ characteristics on the contaminant DNA.

**Table 1:** Specimens subjected to 454-FLX pyrosequencing, number of reads and ratio of endogenous and contaminant sequences obtained.

Specimen	N reads	% endogenous seqs	% human seqs
Neandertal	155,676	0.32% (N = 503)	*
Neolithic	168,998	0.66% (N = 1,117)	*
Lynx	361,151	0.11% (N = 414)	1.4% (N = 5,078)
Myotragus	96,357	0.27% (N = 260)	0.35% (N = 337)

*: in the case of human samples, it is impossible to discern *a priori* which sequences are endogenous and which are human contaminants. However, we have mitochondrial DNA estimates of the maximum potential contamination <1% in the Neandertal sample and <5% in the Neolithic sample.

**Figure 4:**
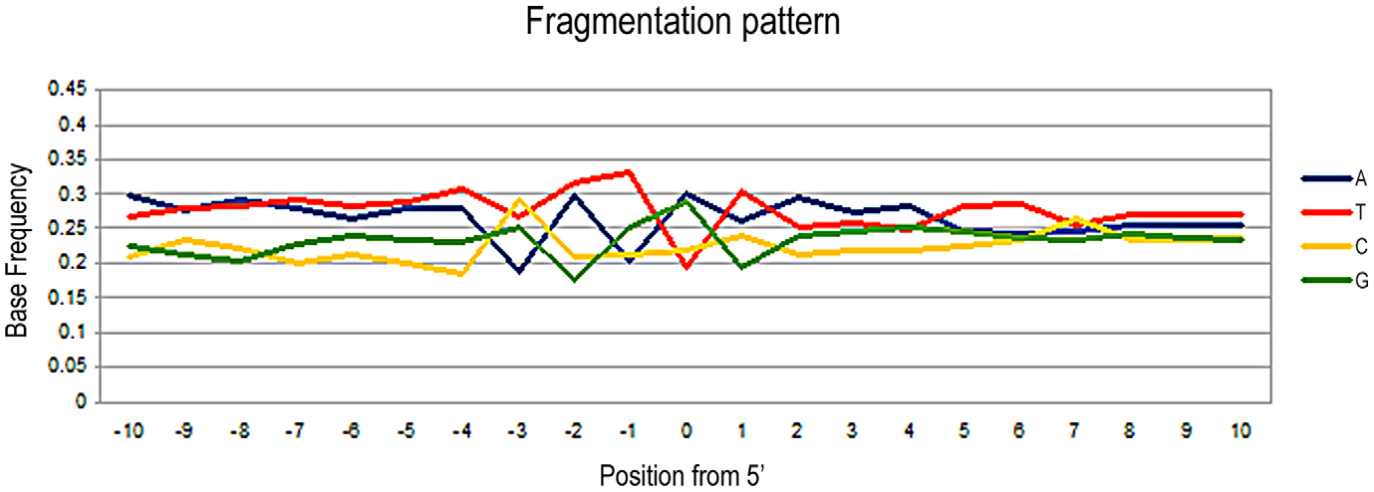
Nucleotide base frequencies at the 5′ end of the human contaminants present in the prehistoric Iberian lynx. The base composition is plotted as a function of distance from the 5′-end (N = 5.078 reads).

Among the 361,151 sequences obtained from the lynx sample, 5,078 (1.4% of the total) were identified as human contaminants, again a figure higher than the endogenous lynx sequences (414, 0.12% of the total reads [Table 1]). In contrast to a previous observation that human contaminant sequences within Neandertal DNA extracts showed no specific increase in the frequency of any nucleotide either side of the DNA fragment [20], the contaminant sequences in the lynx sample show a more complex, non-random fragmentation pattern that is hard to interpret (Figure 4). The pattern at the 5′ end shows an increase of G (instead of the expected T) at the breaking point, of T in subsequent 3′ positions, and an increase of T and A in positions prior to the breaking point (Figure 4). Despite the apparent complexity of the pattern, the resulting entropy is flatly distributed along the breaking point (Figure 3).

### Neolithic sequences

In the Neolithic sample, 168,998 sequence reads were generated, of which 1,117 (0.66%) were human sequences (Table 1). The efficiency of DNA retrieval is again low (<1%), but it is consistent with that obtained from the Myotragus sample that preserved at a similar thermal environment and age [23]. The level is also similar to those previously reported in older specimens, including some Neandertals [14], [19].

**Figure 5:**
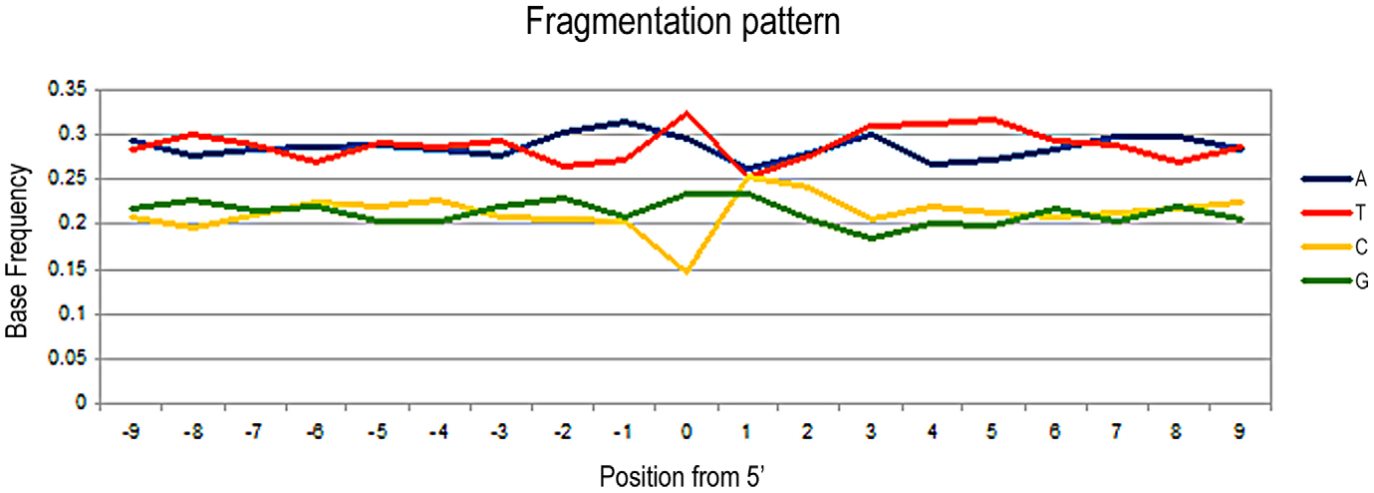
Nucleotide base frequencies at the 5′ end of the human Neolithic sequences. The base composition is plotted as a function of distance from the 5′-end. The depurination-based pattern can be seen, despite the small sample size (N = 1,117 reads).

Among the human sequences, thymine residues at the 5′-ends appear in 32.8% of the DNA fragments, while guanines at the 3′-ends appear as adenines in 28.3% of the cases, Both nucleotides are significantly increased respective of their average frequency along the sequences (Fisher's exact test, P<0.05). Immediately prior to the 5′ strand break, purine bases are elevated to 52.0% (P<0.05), while pyrimidines are decreased to 47.9% (P<0.05). The converse pattern is observed following the 3′-end of the sequence, that is, pyrimidines are increased to 51.9% (P<0.05), while purines are depressed at 48.1% (P = 0.05) (Figure 5). It should be remembered that the 3′-ends correspond to the terminal 5′-position in the complementary strand, and that the whole pattern is consistent with fragmentation at purine sites [17]. As in *Myotragus* sequences, entropy decreases at the breaking point (Figure 3).

### Neandertal sample

**Figure 6:**
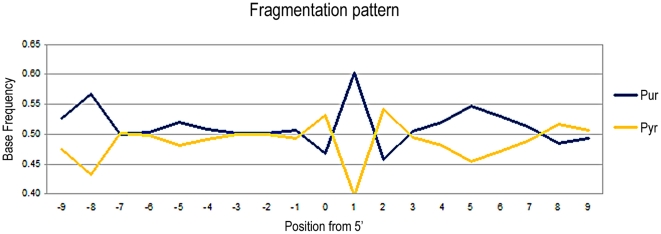
Purine and pyrimidine frequencies at the 5′ and 3′ end of the Neandertal sequences. A–G and C–T have been grouped because of the low number of sequences available (N = 503). Nevertheless, the purine-associated fragmentation pattern can be seen.

In the El Sidrón Neandertal sample, 155,676 reads were generated, of which 503 (0.32%) were primate sequences (Table 1). The efficiency of DNA retrieval is again very low (<1%), but is almost identical (0.27%) to that previously reported from another sample recovered from the same site [19]. The purine fragmentation pattern previously described in ancient sequences can also be seen, although the sample size is lower than that analysed in previous Neandertal studies (N = 503 reads). To visualize the pattern more clearly, we have pooled purine and pyrimidine frequencies (Figure 6). We observe that the frequency of purine residues at the nucleotide immediately prior to the 5′-end are over-represented (56.4%), up from 49.8% at the −1 position. The −1 position is marked by a high decrease of pyrimidines, where only 16.3% of residues in that position are cytosines, followed by a marked increase of them in the position +1 with a 29.9% of all fragments. At the 3′-end the opposite effect can be seen (data not shown). In this case, the breaking point is not marked by a drop in the entropy value (Figure 3), although this may be an artefact of the small sample size. However, considering that the depurination pattern is clearly observed in previously analysed Neandertals [17], it is obvious that the drop in entropy would be also present.

## Discussion

The level of endogenous DNA within these four samples, all from temperate environments but of different ages (ranging between 49,000 and 5,500 years ago) and from heterogeneous taphonomic conditions, are similar to each other, and generally lower (<1%) than the levels observed in previously samples from colder environments [12], [26]–[28]. We note that the non-human samples show considerable human contaminant DNA content (0.35% in *Myotragus* and 1.4% in the lynx), that in both cases outnumber the endogenous sequences, demonstrating in particular the difficulties of working with ancient human specimens, especially those without a detailed handling history. Given this it is clear how analysis of remains that were recently excavated under controlled conditions confers significant benefits. Nevertheless despite this limitation, the given the potential of high-throughput DNA sequencing platforms, it should still be possible to retrieve ancient human genomes from temperate environments.

The sequence size distribution analyses demonstrate that endogenous and contaminant sequences can display different distributions (Figure 1) - the length distributions are significantly different (Kruskal-Wallis test 1095.29, 3 d.f., P<2.2×10^−16^). Obviously, the pattern of size reduction could be related to factors such as the age of the sample or contamination ratios. Thus our data confirms the previous observation that ancient DNA is in general fragmented to smaller lengths than contaminants [4] is confirmed. Despite this, however, we believe that the length of a particular set of sequences is not a helpful authentication criterion per se, due to the wide overlapping of the length distributions in different samples.

The human sequences from the Neolithic and Neandertal sample, as well as the caprine and carnivorous sequences from the *Myotragus* and the lynx, respectively, show the purine-associated fragmentation pattern previously described as a feature of endogenous Neandertal sequences (that is, an elevated level of purines directly prior to the sequence breakpoint, as well as increase of thymines at the 5′ end of sequences and of adenines at the 3′ ends of sequences). We further note that the depurination-based pattern is always stronger at the 5′ end, likely because a number of sequences without the adaptor at the 3′ end were discarded during the analysis.

The data further suggests that contaminant molecules in samples that have been bleached appear to be fragmented following depurination processes, to yield a damage profile that is identical to that exhibited by truly ancient sequences. Until this observation is confirmed or refuted in other samples, we strongly recommended not to bleach human bones that are subsequently to be shotgun-sequenced, because the resulting contaminant sequences will likely imitate the ancient ones in their fragmentation patterns.

In the unbleached samples the human contaminant sequences show nucleotide frequency distributions at the sequences break-points that are different to the depurination-based pattern observed in endogenous sequences, [20]. We speculate that this complex pattern could be the result of the contaminant sequences being fragmented by bacterial enzymes. Further research in this issue is needed, especially in the comparison between recent and old (>10 years) contaminants in a given sample with a known handling story. If entropy parameters are considered, the contaminants do not show a decrease in entropy at the breaking point, in contrast to the known endogenous sequences (with the exception of the Neandertal data, likely due to the small size of the data). Thus, we recommend consideration of entropy as a measure for helping determine whether a given high-throughput sequence dataset derives from contaminant or endogenous sequences.

In conclusion, our results demonstrate that samples preserved under a range of different environmental and taphonomic conditions, over different thermal ages, consistently produce the previously described [17] depurination pattern. Thus, fragmentation patterns are not exclusive of Neandertals or other Late Pleistocene samples, and can be applied as a measure of authenticity across all ancient high-throughput sequencing datasets with increased confidence. The results also confirm that contaminant molecules show neither a simple depurination-associated pattern, nor random nucleotide frequencies along the sequences' lengths, as previously suggested. Furthermore, our results suggest that both sets of sequences (endogenous and contaminants) are not easily distinguishable based on the nucleotide pattern prior to break alone. However, the combination of different statistical tools, including the measure of the entropy at the reads' ends and the distribution lengths, can help to authenticate shotgun sequencing results in the future. Further research on different types of samples (both contaminated and uncontaminated) is needed, since the understanding of the fragmentation patterns is crucial for the human palaeogenomics field.
